# Evaluation of NACA and diNACA in human cystinosis fibroblast cell cultures as potential treatments for cystinosis

**DOI:** 10.1186/s13023-022-02367-w

**Published:** 2022-06-16

**Authors:** Emma Hector, Donald Cairns, G. Michael Wall

**Affiliations:** 1 Robert Gordon University, Aberdeen, UK; 2 Nacuity Pharmaceuticals, Inc., Fort Worth, TX USA

**Keywords:** N-acetylcysteine amide, NACA, diNACA, Cystinosis, Cystine, Cysteine, In vitro, Human fibroblast

## Abstract

**Background:**

Cystinosis is a rare autosomal recessive lysosomal storage disease, associated with high morbidity and mortality. Mutations in the *CTNS* gene disable a membrane protein responsible for the transport of cystine out of the lysosome. Loss of transporter function leads to intralysosomal cystine accumulation and long-term damage to various tissues and organs, including the kidneys, eyes, liver, muscles, pancreas, and brain. The only cystine-depletion therapy for treatment of cystinosis is cysteamine which requires frequent administration of high doses and often causes gastrointestinal pain as well as pungent sulfurous odor in patients. The current in vitro study evaluated antioxidants, N-acetylcysteine amide (NACA; NPI-001) and (2R,2R′)-3,3′-disulfanediyl bis(2-acetamidopropanamide) (diNACA; NPI-002), as potential treatments for cystinosis.

**Methods:**

Cytotoxicity of cysteamine, NACA and diNACA was evaluated in cultured human cystinotic fibroblasts (HCFs). HCFs were cultured in 96 well plates incubated for 0–72 h in the presence of 25, 50 or 75 μM each of either cysteamine, NACA or diNACA along with an untreated control. Media was removed and cell viability assessed. Next, cystine-depleting activities of cysteamine, NACA and diNACA were screened in HCFs cell culture utilizing an inexpensive, proven colorimetric assay. HCFs were seeded and allowed to reach approximately 80% confluence before the addition of the test articles: 50 μM of either cysteamine, NACA or diNACA in media along with an untreated control. HCFs were incubated, harvested, and cystine was reduced to cysteine, the concentration of which was then determined per quantity of protein compared to a cysteine standard. Statistically significant cystine depletion was determined by paired t-test versus untreated control (*p* < 0.05).

**Results:**

Neither cysteamine, NACA nor diNACA at 25, 50 or 75 μM caused cytotoxicity in HCFs. Treatment with all tested concentrations (25, 50 or 75 µM) of either NACA or diNACA at 48 or 72 h resulted in statistically significant increases in cell viability, relative to untreated control, whereas the higher concentrations (50 or 75 µM) of cysteamine achieved statistical significance at both timepoints but not the lowest concentration (25 µM). All test articles depleted cystine from HCFs compared to control. NACA depletion of cystine was statistically superior to cysteamine at 6, 24 and 48 h and numerically greater at 72 h. DiNACA depletion of cystine was statistically superior to cysteamine at 6 and 48 h, slightly numerically greater at 24 h and slightly less at 72 h.

**Conclusions:**

NACA and diNACA were non cytotoxic to HCFs and significantly increased cell viability. Cystine reduction was determined as percent of control after incubation with 50 µM of NACA, diNACA or cysteamine in HCFs cell culture for 6, 24, 48 and 72 h. Of the three test articles, NACA exhibited most rapid and greatest potency in cystine reduction. Rank order potency for cystine reduction over time was observed, NACA > diNACA ≥ cysteamine. Therefore, further study of NACA and diNACA as potential treatments for cystinosis is warranted.

## Introduction

Cystinosis is a rare autosomal-recessive lysosomal storage disease, associated with high morbidity and mortality [1] caused by mutations in the *CTNS* gene located on chromosome 17p13. A number of mutations have been reported, the most common of which is a 57 Kb deletion present in approximately 50% of cystinotic patients of Western European ancestry [18]. *CTNS* codes for the cystine transporter, cystinosin, a 367 amino acid integral membrane protein responsible for the transport of cystine out of the lysosome. Loss of transporter function therefore leads to intralysosomal cystine accumulation, and long-term damage to various tissues and organs, including the kidneys, eyes, liver, muscles, pancreas, and brain [33].

Cystinosis comprises three major phenotypes: (1) nephropathic cystinosis in untreated children is characterized by renal Fanconi syndrome, poor growth, hypophosphatemic/calcipenic rickets, impaired glomerular function resulting in complete glomerular failure, and accumulation of cystine in almost all cells, leading to cellular dysfunction with tissue and organ impairment. The typical untreated child has short stature, rickets, and photophobia. Failure to thrive and signs of renal tubular Fanconi syndrome (polyuria, polydipsia, dehydration, and acidosis) typically appear as early as age 6 months; corneal crystals can be present before age one year and are always present after age 16 months. Prior to the use of renal transplantation and cystine-depleting therapy, the life span in nephropathic cystinosis was seldom longer than ten years. With these interventions, affected individuals can survive at least into the mid-forties or fifties with satisfactory quality of life; (2) intermediate cystinosis is characterized by all the typical manifestations of nephropathic cystinosis, but onset at a later age. Renal glomerular failure occurs in all untreated affected individuals, usually between ages 15 and 25 years; and (3) non-nephropathic (ocular, corneal) form of cystinosis characterized clinically only by photophobia resulting from corneal cystine crystal accumulation. Corneal cystine crystal accumulation is a common manifestation in all cystinosis patients [16]. Diagnosis and management have been reviewed elsewhere [21].

All approved drugs for cystinosis contain the aminothiol, cysteamine. Cysteamine treatment causes intralysosomal formation of a cysteamine–cysteine mixed disulfide, capable of exiting the lysosome via the lysine transport pathway [7, 32]. CYSTAGON® and PROCYSBI® are immediate- and delayed-release, respectively, oral dosage forms of cysteamine bitartrate. Oral administration of cysteamine, although efficacious for nephropathic cystinosis, is associated with poor patient compliance due to the unpalatable taste and odor of sulfur, gastrointestinal side-effects such as nausea, vomiting and pain, coupled with a requirement for frequent, high dosing to overcome extensive first pass metabolism of the cysteamine [12]. Furthermore, oral cysteamine therapy does not reach the avascular corneal tissues so is ineffective in treating ocular (corneal) cystinosis [10]. Similarly to oral dosage forms, CYSTARAN® and CYSTADROPS® eye drops, both containing cysteamine hydrochloride, require frequent dosing, and cause discomfort. Hence, there is an unmet medical need for alternative treatments for cystinosis.

**Fig. 1:**
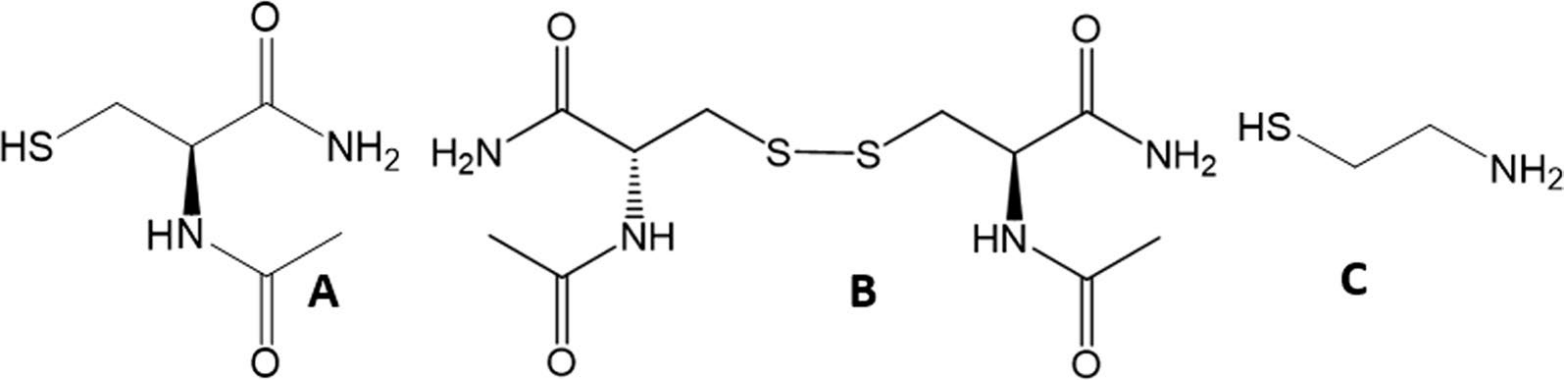
**A** N-acetylcysteine amide (NACA, NPI-001); **B** (2R,2R′)-3,3′-disulfanediyl bis(2-acetamidopropanamide) (diNACA, NPI-002); **C** cysteamine

The current study describes the evaluation of anticystinotic activity of novel compounds, N-acetylcysteine amide (NACA, NPI-001) and (2R,2R′)-3,3′-disulfanediyl bis(2-acetamidopropanamide) (diNACA, NPI-002), compared to cysteamine (Fig. 1), with a view to determining whether these compounds may be safe and sufficient to reduce cystine and thereby effectively treat nephropathic or ocular cystinosis.

## Materials and methods

### Materials

N-acetylcysteine amide (NACA; NPI-001) and (2R,2R′)-3,3′-disulfanediyl bis(2-acetamidopropanamide) (diNACA; NPI-002) were provided by Nacuity Pharmaceuticals, Inc., Fort Worth, Texas, USA. Human cystinotic fibroblasts (HCFs) were obtained from a commercial laboratory (GM00008, Coriell Cell Repositories, NJ, USA). Eagle’s minimum essential media supplemented with 15% FBS, 200 U/ml penicillin, 200 μg/ml streptomycin and 2 mM glutamine was obtained from Thermo Fisher Scientific, UK. Bovine serum albumin (Thermo Fisher Scientific, UK), Bradford Reagent (Sigma Aldrich, UK), cysteamine HCl (Sigma Aldrich, UK), dimethyl sulfoxide (DMSO; Thermo Fisher Scientific, UK), 3-(4,5-dimethylthiazol-2-yl)-2,5-diphenyltetrazolium bromide (MTT) (Sigma Aldrich, UK), N-benzoyl-L-arginine p-nitroanilide (L-BAPNA) and Molecular Probes’ Thiol and Sulfide Quantitation Kit (Thermo Fisher Scientific, UK), N-ethylmaleimide (Sigma Aldrich, UK), carbon dioxide gas (CO_2_; BOC Gases, UK), liquid nitrogen (BOC Gases, UK), NaBH_4_ (Sigma Aldrich, UK), NaOH (Sigma Aldrich, UK), and Papain-SSCH_3_ (Thiol and Sulfide Quantitation Kit component; Thermo Fisher Scientific, UK) were obtained from reputable sources.

### Cystinotic fibroblast cell culture

HCFs were cultured in Eagle’s minimum essential media supplemented with 15% FBS, 200 U/ml penicillin, 200 μg/ml streptomycin and 2 mM glutamine, in a humidified atmosphere at 37 °C and 5% CO_2_. Cysteine/cystine have been reported as common contaminants in culture media [25]. The culture media used herein was not modified to be cystine-free. Because all cell culture conditions, apart from the application of test article, were the same, any interference from traces of cystine from the growth media would be consistent across all samples. Cells were routinely maintained in 75 cm^2^ flasks prior to harvesting and use in the following experiments. All experiments were carried out under these growth conditions using the media mixture described above, supplemented with test articles.

### Cytotoxicity of NACA and diNACA

Concentration ranges of NACA and diNACA incubated along with HCFs in media were based on typical cysteamine peak plasma concentration achieved (range of 30–70 µM) following an approximate therapeutic dose of 15 mg/kg cysteamine bitartrate [28]. HCFs were cultured in 96-well plates incubated for 48 and 72 h in the presence of 25, 50 or 75 μM each of either cysteamine (as HCl salt), NACA or diNACA. Media was removed and cells were incubated in 0.5 mg/ml of MTT reconstituted in media. A colorimetric assay used reduction of the yellow tetrazolium salt (MTT) to a purple formazan salt to measure cellular metabolic activity as a proxy for cell viability [35]. Treated cells were incubated in MTT for 4 h at 37 °C, after which time intracellular purple formazan salt crystals were visible under a microscope. MTT solution was then removed, and DMSO was added to each well to lyse cells and dissolve the salt crystals. After 1 h incubation at 37 °C, absorbance was measured on a multiwell plate reader (Biotek FL6000) at 570 nm, and percentage cell viability calculated [35].

### Cystine-depleting activity of NACA and diNACA

**Fig. 2:**
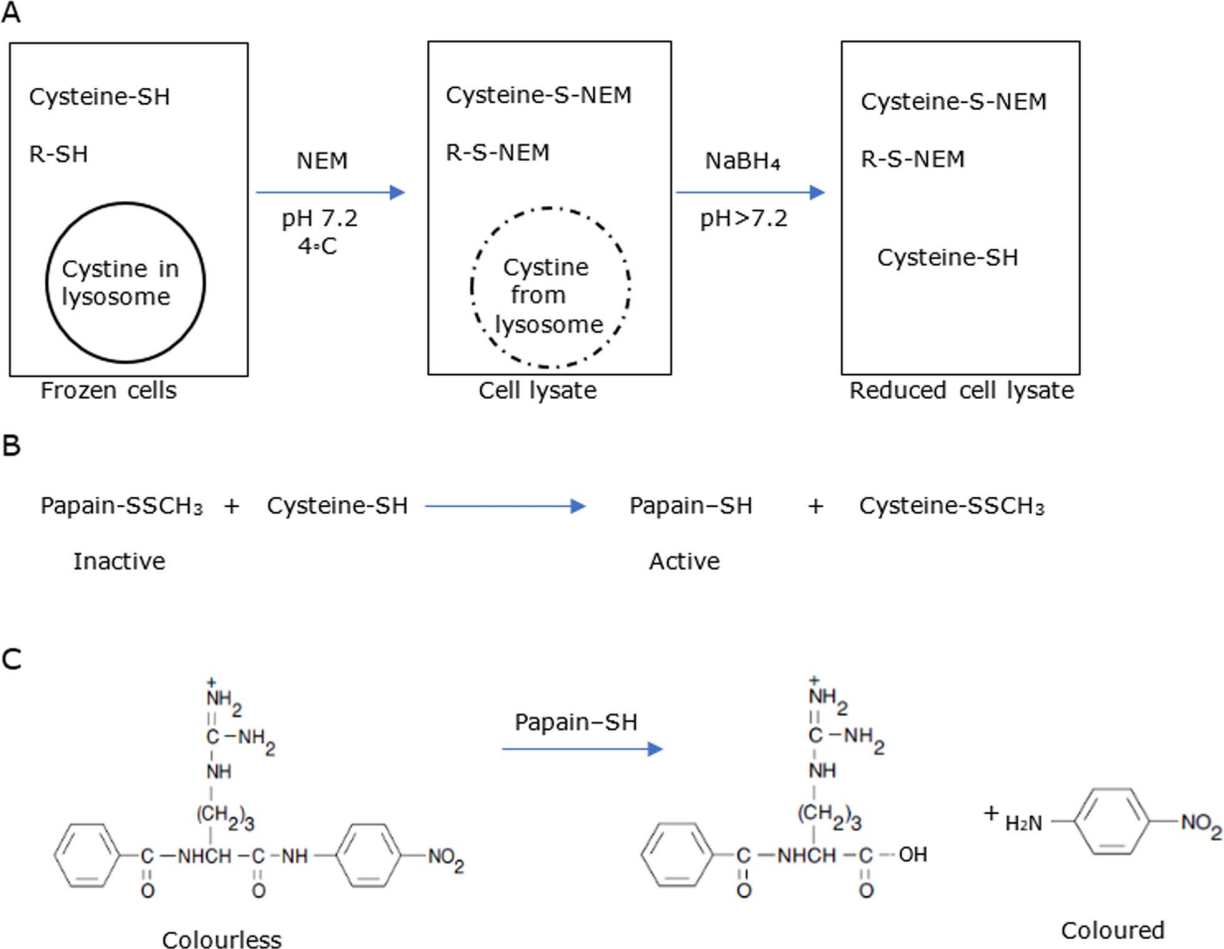
Cystine quantification method utilized herein: **A** N-ethylmaleimide (NEM) blocked non-cystine thiols before cell lysis; cystine lysed from lysosome was reduced by NaBH_4_ to cysteine. **B** The inactive disulfide derivative of papain was activated in the presence of cysteine. **C** Papain cleaves the substrate N-benzoyl-L-Arginine-4-nitroanilide hydrochloride (L-BAPNA) releasing the p-nitroaniline chromophore detectable at 410 nm. Values were quantified by comparison to a cysteine standard. Quantity of cysteine was therefore stoichiometrically equal to p-nitroaniline thus serving as a surrogate for lysosomal cystine concentration

Lysosomal cystine was converted to cysteine, adapted from works by de Graf-Hess et al. [9], further refined by McCaughan et al. [19], and successfully utilized by Omran et al. [22–24] (Fig. 2). Molecular Probes’ Thiol and Sulfide Quantitation Kit provided an ultrasensitive colorimetric assay for quantitating cysteine. In this assay, which is based upon a reported method [26, 27], thiols or inorganic sulfides reduce a disulfide-inhibited derivative of the enzyme, papain, stoichiometrically releasing the active enzyme. The activity of the enzyme, papain, is then measured using the chromogenic papain substrate, L-BAPNA. Although thiols and inorganic sulfides can also be quantitated using the traditional Ellman’s reagent (5,5′-dithiobis-[2-nitrobenzoic acid]), the enzymatic amplification step in Molecular Probes’ kit enables a detection limit 0.2 μM thiol (0.2 nmol in a 1 mL reaction), a sensitivity that is about 100-fold greater than that achieved using Ellman’s reagent.

HCFs were seeded in a 25 cm^2^ vented flask and allowed to reach approximately 80% confluence before the addition of the test articles: 50 μM of either cysteamine (as HCl salt), NACA or diNACA in 4 cm^3^ culture media for incubation periods of 6, 24, 48 and 72 h. The cells were harvested, washed three times in phosphate-buffered saline, flash-frozen in liquid nitrogen and stored at − 80 °C until the relative cysteine concentration was determined per quantity of protein. The cells were recovered from storage at − 80 °C and suspended in 100 μL 1 mM N-ethylmaleimide prepared in phosphate buffer (pH 7.2) followed by sonication for 10 s which was repeated 3 times with 20 s cooling intervals on ice. The solution was centrifuged at 800 G for 10 min at 4 °C (Biofuge primo R Heraeus centrifuge). To this cell supernatant (40 μl) was added 4 μl of 4 M NaBH_4_ in 7:3 0.1 M NaOH/DMSO. After 5 min incubation at room temperature, 800 μl of sodium acetate buffer (pH 4.7) was added. A 5 μl volume of the diluted solution was added to 100 μl of 0.6 mg/ml solution of Papain-SSCH_3_ in a 96 well plate and incubated for one hour at room temperature. A 100 μl volume of 4.9 mM BANI solution in sodium acetate buffer (pH 4.0) was added to each well of the 96 well plate, gently mixed and incubated for further one hour at room temperature. Absorption at 410 nm was measured and the cystine levels were calculated by comparison to known cystine standard.

The protein concentration in every sample was measured according to Bradford method. Briefly, 200 μl of Bradford reagent was added to 5 μl of cell supernatant in each well of a 96 well plate and incubated for 5 min at room temperature and the absorption at 595 nm was measured. Protein concentration was calculated by extrapolation from a standard curve of 0–1.0 mg/ml bovine serum albumin. Cystine values, determined by the thiol detection assay as μM cysteine by comparison to the known cysteine standards, were then divided by the protein concentration of the sample (mg) to normalize cystine concentration. This was expressed as percentage of the control (untreated cells) for each test agent. Statistically significant cystine depletion was determined by paired t-test versus untreated control (*p* < 0.05).

## Results

### NACA and diNACA Lacked Cytotoxicity and Increased Viability of HCFs

**Fig. 3:**
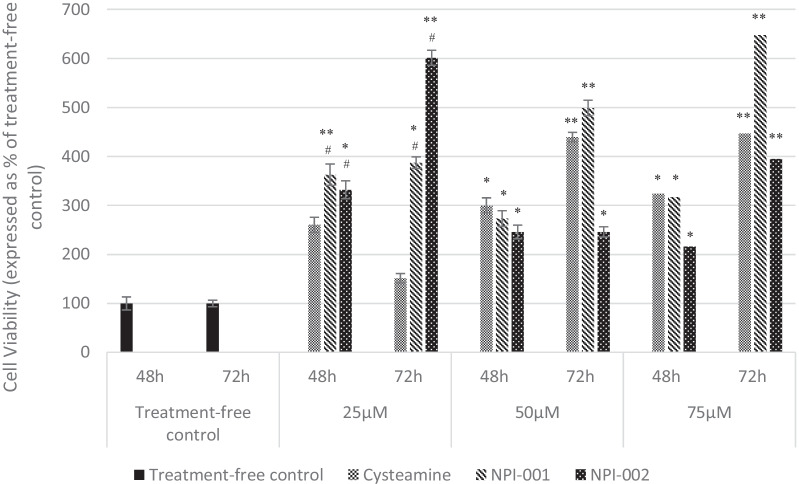
HCFs viability (expressed as percentage of treatment-free control) following incubation with 25, 50 or 75 µM cysteamine, NPI-001 (NACA) or NPI-002 (diNACA) at 48 and 72 h. Data shown are the mean of 5 independent measurements, ± S.E.M. Level of significance at 48 and 72 h was determined by paired t-test versus the respective treatment-free control (*p < 0.05, **p < 0.005) or respective cysteamine treated sample (#*p* < 0.05)

Exposure to 25, 50 or 75 µM concentrations of NACA, diNACA or cysteamine for 48 or 72 h caused no cytotoxicity in HCFs. Moreover, treatment with 25, 50 or 75 µM of either NACA and diNACA at 48 or 72 h resulted in statistically significant increases in cell viability, relative to untreated control. At the greatest concentrations (50 or 75 µM), cysteamine achieved statistical significance at both timepoints but not at the lowest concentration (25 µM) (Fig. 3).

### Cystine-depleting activity of NACA and diNACA

**Fig. 4:**
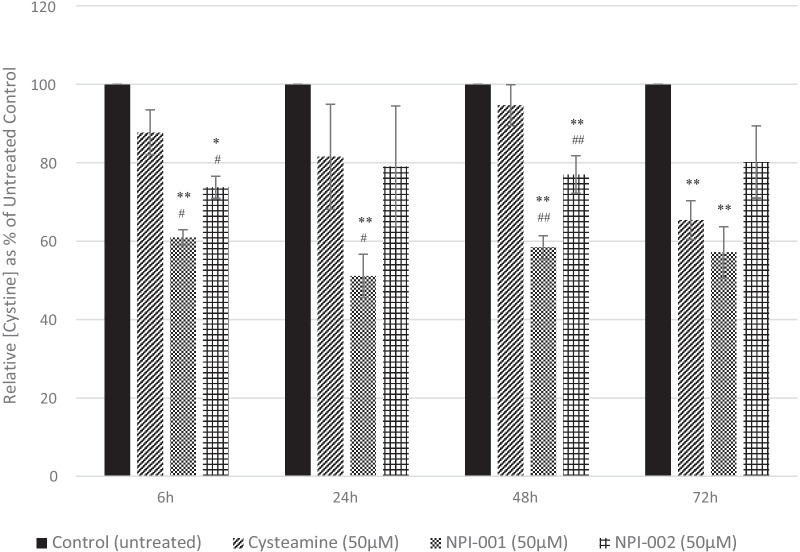
Relative cystine depletion in HCFs (expressed as percentage of treatment-free control) measured after 6, 24, 48- and 72-h incubation with cysteamine, NPI-001 (NACA) or NPI-002 (diNACA) (all at 50 µM). The data shown for each time point is the mean of 1–3 independent experiments, with each independent experiment measured in triplicate ± S.E.M. Level of significance was determined by paired t-test versus the relative untreated control (*p < 0.05, **p < 0.005) and versus the relative cysteamine treated sample (#*p* < 0.05, ##*p* < 0.005) for each time point

Cystine reduction was determined as percent of control after incubation of NACA, diNACA or cysteamine in HCFs cell culture for 6, 24, 48 and 72 h. Of the three test articles, NACA exhibited most rapid and greatest potency in cystine depletion. NACA induced statistically significant cystine reductions versus control at all time points. NACA was statistically more potent than cysteamine at earlier timepoints (6, 24, 48 h) and numerically, but not statistically, superior at 72 h. DiNACA was statistically superior to cysteamine at 6 and 48 h, marginally more potent at 24 h and slightly less at 72 h. DiNACA significantly reduced cystine at 6 and 48 h and numerically reduced cystine at 24 and 72 h. Cysteamine numerically reduced cystine at 6, 24 and 48 h, reaching statistical significance at 72 h. Rank order potency for cystine reduction over time was observed: NACA > diNACA ≥ cysteamine (Fig. 4).

## Discussion

Cysteamine is the only approved drug treatment specifically for cystinosis. In cystinosis patients, cysteine is oxidized to cystine that accumulates in the lysosome. Cysteamine enters the lysosome via an unknown transport mechanism where it breaks the disulfide bond in cystine, leading to the formation of cysteine and cysteine–cysteamine disulfide [13], and the resulting mixed disulfide leaves the lysosome possibly via the lysine transporter [7]. Several cysteamine derivatives or prodrugs have been reported to have favorable activity profiles, shown to be as, or even more, effective than cysteamine in depleting intralysosomal cystine [5, 6, 11, 19, 22–24, 28].

Other thiols have been assessed for their anticystinotic activity. A seminal paper by Thoene et al. [31] explored structure-activity relationships of several small molecule amines, thiols and aminothiols with regard to cystine-depletion activity. They reported that 100 µM cysteamine rapidly depletes cystine from HCFs to undetectable levels in 1 h: cystamine (the oxidation product of cysteamine) produced only a 35% decrease and glutathione (GSH) produced no decrease. The dimethyl derivative, 2-dimethylaminoethanethiol at 1 mM, was approximately as effective as cysteamine in reducing intracellular nonprotein cystine. At these concentrations and reaction conditions, sulfhydryl compounds of similar mass but possessing a hydrogen, hydroxyl, or methyl group instead of an amine in the 2-position (adjacent to thiol) did not deplete cystine from HCFs. Dithiols, 1,3-propanethiol and dithiothreitol, reduced cystine by nearly 50% at 1 h. Ethanolamine, with a mass similar to cysteamine but without a sulfhydryl moiety, produced no cystine depletion either in cells or in the simple chemical reaction. Cystamine, the disulfide of cysteamine, was highly effective in reducing cystine in HCFs but ineffective in lowering the cystine concentration in a cell-free system: presumably it requires reaction with GSH or GSH reductase to yield cysteamine for activity. The monosulfide, bis(2-aminoethyl) sulfide, did not reduce cystine in either system. Penicillamine was ineffective in reducing cystine. Diffusion through lysosomal membranes is typically restricted to molecules of mass less than 200–230 daltons [31]. Also, Taranta et al. [30] reported the cystine-depleting and anti-apoptotic properties of the disulfide, disulfiram, confirmed by secondary in vitro assays and after treating Ctns–/– mice with 200 mg/kg/day of disulfiram for 3 months. However, at this dosage, growth impairment was observed and long-term treatment with a lower dose (100 mg/kg/day) did not inhibit growth, but failed to reduce cystine accumulation, caused premature death, and did not prevent the development of renal lesions.

Previous studies suggested that the increased oxidative stress plays a role in the pathogenesis of cystinosis [14, 34]. Indeed, the antioxidant, N-acetylcysteine (NAC, the active ingredient in MUCOMYST®, ACETADOTE® and CETYLEV®) has been shown to improve cystinosis. The authors of a 3-month study of 23 cystinosis patients less than 18 years old without renal replacement therapy, concluded that the addition of oral NAC 25 mg/kg/day to cysteamine standard of care caused a reduction in oxidative stress, based on a decrease in serum thiobarbituric acid-reactive substances (TBARS) (p < 0.0001), an improvement in renal function (based on levels of serum cystatin C), creatinine levels, and creatinine clearance [14].

As reported, NACA, the amide derivative of NAC, reduces oxidative stress in numerous disease models [29]. The major metabolite of NACA is NAC but NACA is more bioavailable (yields nearly double systemic [NAC] in mouse compared to NAC alone) than NAC [15], so, NACA may be considered an efficient prodrug of NAC. Furthermore, the GSH-replenishing capacity of NACA is three- to four-fold greater after the administration of NACA compared to NAC in mice [15]. And the dimer of NACA, diNACA, has been shown to serve as a prodrug to NACA and NAC [36]. Both NACA and diNACA were shown to protect skin cell cultures from oxidative stress conditions [20]. Hence, NACA and diNACA were evaluated as potential anticystinotic agents herein.

Results of the current study have demonstrated that NACA and diNACA lack cytotoxicity and even enhance growth of HCFs. And NACA and diNACA are effective in cystine reduction in HCFs.

Selection of a reliable quantification method for cystine depletion herein was a key issue. For this initial screening study of NACA and diNACA, the authors sought an economical, reliable, quick test of cystine depletion compared to cysteamine. To establish, optimize and qualify a liquid chromatographic-mass spectrometry assay would have been a time-consuming and expensive exercise, and the absolute value of cystine was not as important as the relative activity of test articles. Therefore, the decision was made to utilize a previously peer-reviewed and published assay method with which the authors were experienced [19, 22–24]. 

A seeming discrepancy of the study herein is that 50 µM cysteamine reduced cystine (about 10% after 6 h, about 40% after 72 h) much less than expected based on previous results for 100 µM cysteamine (nearly 100% reduction in 1 h; [31]). This may be because culture media used herein was not modified to be cystine-free. There could have been interference from traces of cystine from the growth media, though, it would have been consistent across all samples regardless of test conditions, relative to control. Alternatively, it is possible that the batch of cysteamine used was less effective than in previous assays, (perhaps through oxidation to cystamine), or perhaps the passage number of the cells was different to those used previously. The concentration of test articles utilized in the current cystine-depletion study (50 µM) was informed by the work of Smolin et al. [28] who determined that peak plasma concentration achieved 1 h following an approximate therapeutic cysteamine bitartrate dose of 15 mg/kg ranged from 30 to 70 µM. Results are therefore not directly comparable to those of Thoene et al. [31] because, in addition to the lower test article concentration, incubation times varied considerably from that employed by the aforementioned study. Time periods of 6, 24, 48 and 72 h provide evidence of the durability of cystine-depleting effect of NACA and diNACA (Fig. 4). For the assay herein, appropriate controls were carried out, and all measurements were made under identical conditions. The results, therefore, are true values, even though the absolute activity of the cysteamine is different from previous, published measurements.

The mechanism of anticystinotic activity of NACA and diNACA requires investigation. The primary thiol, NACA may, like cysteamine, react with cystine to form a mixed disulfide. In addition to the current data, evidence for this includes the finding during the development of analytical methods for total NACA in tissues, a reagent was needed to reduce NACA-mixed disulfides that spontaneously formed in tissues [17]. Whether diNACA acts through a mixed-disulfide mechanism (analogous to cystamine, based on Theone et al., [31]) is also of interest.

Currently, both NACA and diNACA are in clinical trials based on their antioxidant properties. An oral dosage form of NACA is the subject of an ongoing clinical trial for the treatment of retinitis pigmentosa (Safety and Efficacy of NPI-001 Tablets for RP Associated with Usher Syndrome (SLO RP) (ClinicalTrials.gov Identifier: NCT04355689)). Though masked, some patients have surpassed one year of treatment with no significant gastrointestinal or odoriferous effects reported. An intravitreal implant containing diNACA is under evaluation as an anticataract treatment (NPI-002 Intravitreal Implant for the Delay of Cataract Progression (ClinicalTrials.gov Identifier: NCT05026632)).

## Conclusions

NACA and diNACA caused no cytotoxicity. Treatment with all tested concentrations (25, 50 or 75 µM) of either NACA or diNACA at 48 or 72 h resulted in statistically significant increases in cell viability, relative to untreated control, whereas the higher concentrations (50 or 75 µM) of cysteamine achieved statistical significance at both timepoints, but not the lowest concentration (25 µM). Cystine reduction was determined as percent of control after incubation with 50 µM of NACA, diNACA or cysteamine in HCFs cell culture for 6, 24, 48 and 72 h. Rank order potency for cystine reduction over time was observed: NACA > diNACA ≥ cysteamine. These findings warrant further evaluation of NACA and diNACA as potential therapies for nephropathic or corneal cystinosis.

## Data Availability

The datasets used and/or analyzed during the current study are available from the corresponding author on reasonable request.
